# Supplementary material for: Left ventricular mass normalization in child and adolescent athletes must account for sex differences
Source: PLoS One. 2020 Jul 27;15(7):e0236632. doi: 10.1371/journal.pone.0236632 (PMC7384656; doi:10.1371/journal.pone.0236632)
Supplement: S1 Table — (DOCX) [file pone.0236632.s005.docx]

**S1 Table. The mean differences between the paired non-specific and sex-specific z-scores in girls and boys.**

|  | **Girls** | **Boys** | ***p-value*** |
| --- | --- | --- | --- |
| LVM for Height (LMS) | -0.3218 (0.2184) | 0.2171 (0.0956) | *p<0.0001* |
| LVM for BSA (LMS) | -0.3725 (0.2654) | 0.2507 (0.0622) | *p<0.0001* |
| LVM for cLBM (LMS) | -0.1670 (0.1608) | 0.1118 (0.0846) | *p<0.0001* |
| LMV indexed to BSA | -0.4611 (0.3209) | 0.3115 (0.0604) | *p<0.0001* |
| LVM indexed to height*^2.7^* | -0.3843 (0.2569) | 0.2596 (0.0662) | *p<0.0001* |
| LVM indexed to BSA*^b^* | -0.4154 (0.2424) | 0.2806 (0.0459) | *p<0.0001* |
| LVM indexed to height*^bs^* | -0.3943 (0.2858) | 0.2664 (0.0730) | *p<0.0001* |

The data are expressed as “mean difference (standard deviation).” LMS in brackets means that these LVM normative data were produced using the LMS method. For BSA*^b^*, the BSA is raised to the power of *b*, where *b* is equal to the allometric exponent estimated for the combined group; for height*^bs^*, the height is raised to the power of *bs*, where *bs* is equal to the allometric exponent that is group-specific - estimated separately for the combined group, for girls, and boys, respectively.
