# Supplementary material for: Left ventricular mass normalization in child and adolescent athletes must account for sex differences
Source: PLoS One. 2020 Jul 27;15(7):e0236632. doi: 10.1371/journal.pone.0236632 (PMC7384656; doi:10.1371/journal.pone.0236632)
Supplement: S2 Table — (DOCX) [file pone.0236632.s006.docx]

**S2 Table. Pearson correlation coefficients and the slopes of the regression lines for relationships between the differences between non-specific and sex-specific z-score and the averages of non-specific and sex-specific z-scores.**

|  | **Pearson Coefficient** | **Slope** |
| --- | --- | --- |
| **Girls** |  |  |
| LVM for Height (LMS) | -0.5312 (p<0.0001) | -0.1232 (p<0.0001) |
| LVM for BSA (LMS) | -0.3989 (p<0.0001) | -0.1124 (p<0.0001) |
| LVM for cLBM (LMS) | -0.5492 (p<0.0001) | -0.0925 (p<0.0001) |
| LMV indexed to BSA | -1.0000 (p=0.0000) | -0.3822 (p – N/A) |
| LVM indexed to height*^2.7^* | -1.0000 (p=0.0000) | -0.2948 (p=0.0000) |
| LVM indexed to BSA*^b^* | -1.0000 (p=0.0000) | -0.2758 (p – N/A) |
| LVM indexed to height*^bs^* | -0.9269 (p<0.0001) | -0.3059 (p<0.0001) |
| **Boys** |  |  |
| LVM for Height (LMS) | 0.1386 (p=0.0021) | 0.0013 (p=0.0021) |
| LVM for BSA (LMS) | 0.1899 (p<0.0001) | 0.0119 (p<0.0001) |
| LVM for cLBM (LMS) | 0.4773 (p=0.0000) | 0.0395 (p=0.0000) |
| LMV indexed to BSA | 1.0000 (p=0.0000) | 0.0587 (p<0.0001) |
| LVM indexed to height*^2.7^* | 1.0000 (p=0.0000) | 0.0640 (p=0.0000) |
| LVM indexed to BSA*^b^* | 1.0000 (p=0.0000) | 0.0448 (p – N/A) |
| LVM indexed to height*^bs^* | 0.8984 (p<0.0001) | 0.0635 (p<0.0001) |

LMS in brackets means that these LVM normative data were produced using the LMS method. For BSA*^b^*, the BSA is raised to the power of *b*, where *b* is equal to the allometric exponent estimated for the combined group; for height*^bs^*, the height is raised to the power of *bs*, where *bs* is equal to the allometric exponent that is group-specific - estimated separately for the combined group, for girls, and boys, respectively.
