# Supplementary material for: Left ventricular mass normalization in child and adolescent athletes must account for sex differences
Source: PLoS One. 2020 Jul 27;15(7):e0236632. doi: 10.1371/journal.pone.0236632 (PMC7384656; doi:10.1371/journal.pone.0236632)
Supplement: S3 Table — (DOCX) [file pone.0236632.s007.docx]

**S3 Table. Pearson correlation coefficients and the slopes of the regression lines for relationships between the LVM z-scores and the corresponding body size variables.**

|  | **Pearson Coefficient** | **Slope** |
| --- | --- | --- |
| **Girls** |  |  |
| LVM for Height (LMS) | 0.0018 (p=0.9734) | 0.0001 (p=0.9734) |
| LVM for BSA (LMS) | 0.0016 (p=0.9763) | 0.0059 (p=0.9763) |
| LVM for cLBM (LMS) | 0.0018 (p=0.9734) | 0.0002 (p=0.9734) |
| LMV indexed to BSA | 0.2582 (p<0.0001) | 0.9262 (p<0.0001) |
| LVM indexed to height*^2.7^* | 0.1742 (p=0.0015) | -0.0117 (p=0.0015) |
| LVM indexed to BSA*^b^* | 0.1941 (p=0.0004) | -0.6963 (p=0.0004) |
| LVM indexed to height*^bs^* | 0.0015 (p=0.9779) | 0.0001 (p=0.9779) |
| **Boys** |  |  |
| LVM for Height (LMS) | 0.0027 (p=0.9523) | 0.0001 (p=0.9523) |
| LVM for BSA (LMS) | 0.0019 (p=0.9666) | 0.0052 (p=0.9666) |
| LVM for cLBM (LMS) | 0.0014 (p=0.9751) | 0.0001 (p=0.9751) |
| LMV indexed to BSA | 0.4429 (p<0.0001) | 1.2034 (p<0.0001) |
| LVM indexed to height*^2.7^* | 0.0620 (p=0.1705) | -0.0031 (p=0.1705) |
| LVM indexed to BSA*^b^* | 0.0212 (p=0.6393) | 0.0577 (p=0.6393) |
| LVM indexed to height*^bs^* | 0.0224 (p=0.6207) | 0.001 (p=0.6207) |
| **Combined groups** |  |  |
| LVM for Height (LMS) | 0.0035 (p=0.9235) | 0.0002 (p=0.9235) |
| LVM for BSA (LMS) | 0.0016 (p=0.9633) | 0.0047 (p=0.9633) |
| LVM for cLBM (LMS) | 0.0001 (p=0.9836) | 0.0001 (p=0.9836) |
| LMV indexed to BSA | 0.4327 (p<0.0001) | 1.2687 (p<0.0001) |
| LVM indexed to height*^2.7^* | 0.0272 (p=0.4358) | -0.0015 (p=0.4358) |
| LVM indexed to BSA*^b^* | 0.0315 (p=0.3668) | 0.0925 (p=0.3668) |
| LVM indexed to height*^bs^* | 0.0242 (p=0.4890) | 0.0013 (p=0.4890) |

LMS in brackets means that these LVM normative data were produced using the LMS method. For BSA*^b^*, the BSA is raised to the power of *b*, where *b* is equal to the allometric exponent estimated for the combined group; for height*^bs^*, the height is raised to the power of *bs*, where *bs* is equal to the allometric exponent that is group-specific - estimated separately for the combined group, for girls, and boys, respectively.
