# Supplementary figures and images for: Left ventricular mass normalization in child and adolescent athletes must account for sex differences
Source: PLoS One. 2020 Jul 27;15(7):e0236632. doi: 10.1371/journal.pone.0236632 (PMC7384656; doi:10.1371/journal.pone.0236632)

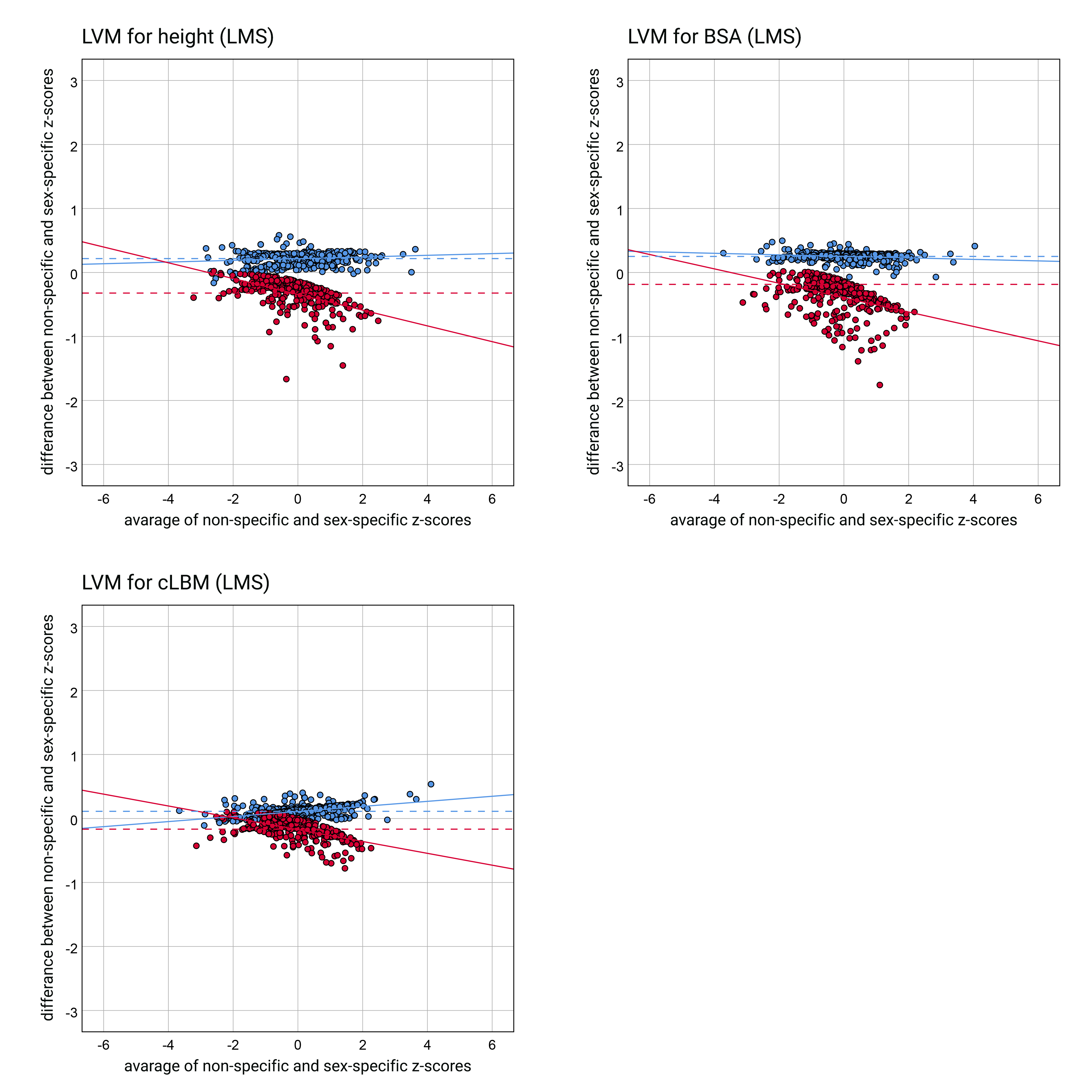

Supplement: S1 Fig — The data points corresponding to girls are red, and to boys are blue. Regression lines are fitted to the data points—the solid red line to girls and the solid blue line to boys. Two horizontal lines corresponding to the mean difference for girls (dashed red line) and boys (dashed blue line) are drawn as well. BSA, body surface area according to Haycock formula [33]; cLBM, lean body mass computed according to Foster’s at al. equations [34]; (TIF) [file pone.0236632.s008.tif]

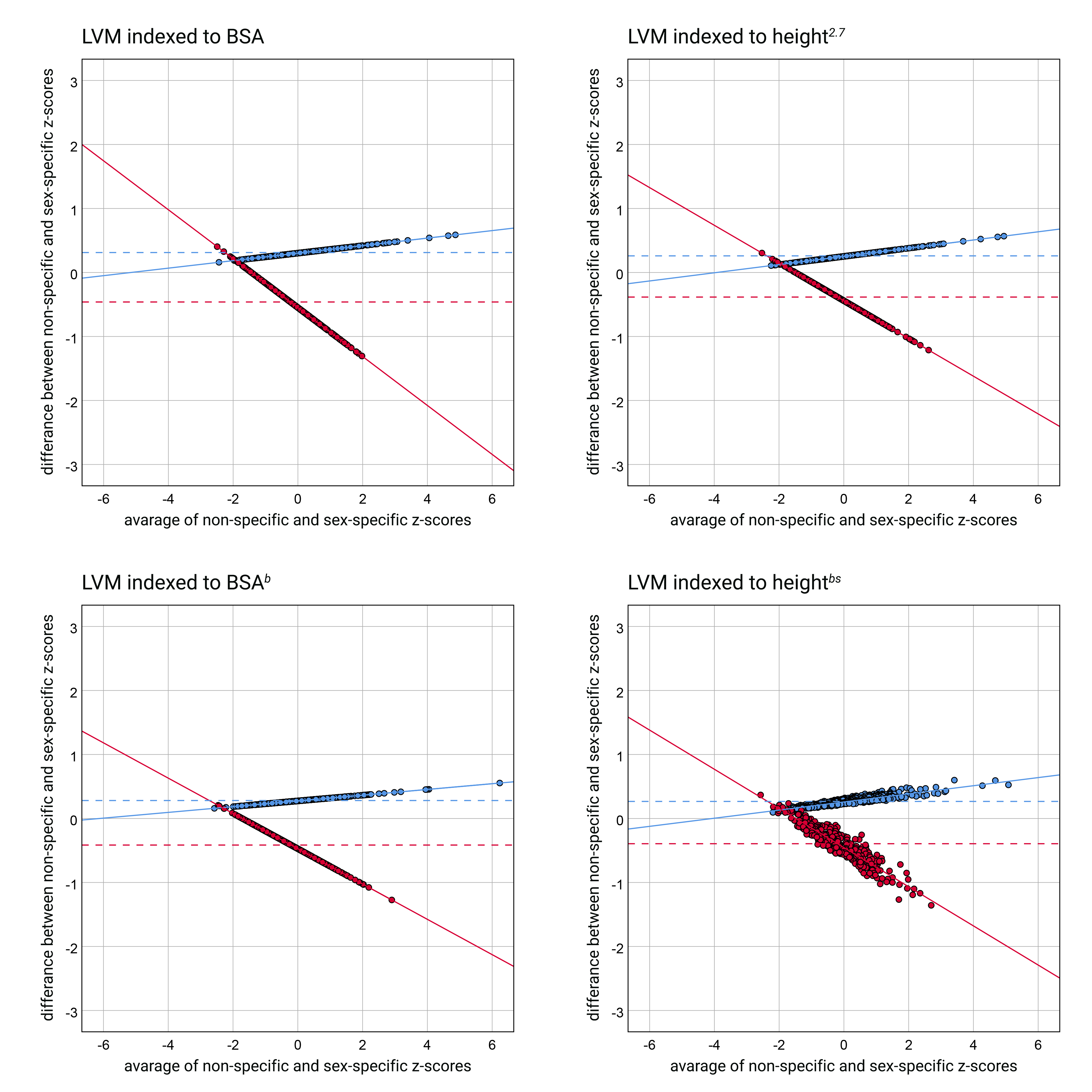

Supplement: S2 Fig — The design of the scatter plots is the same as for S1 Fig. (TIF) [file pone.0236632.s009.tif]
