# Supplementary material for: Left ventricular mass normalization in child and adolescent athletes must account for sex differences
Source: PLoS One. 2020 Jul 27;15(7):e0236632. doi: 10.1371/journal.pone.0236632 (PMC7384656; doi:10.1371/journal.pone.0236632)
Supplement: S1 Text — They picture the overestimation of relative LVM in boys and underestimation in girls when the LVM normative data that are not sex-specific are used. (DOCX) [file pone.0236632.s010.docx]

**Examples of LVM z-score calculations**

The examples picture overestimation of relative LVM in boys and underestimation in girls when the LVM normative data that are not sex-specific are used.

1. Computation of LVM z-score for an individual child, from the L, M, and S values corresponding to the child’s height.

**Child 1**: 15-year-old girl, 166 cm in height, with actual LVM = 162.1156 g (observation number 282 in the S4 Dataset). The LVM z-score calculations are made according to equation (1), based on the respective L, M, and S values corresponding to her height, as follows:

| (1) | $z\text{-}score=\frac{\left[ \left( \frac{actual LVM}{M} \right)^{L}-1 \right]}{L\times S}$ | |
| --- | --- | --- |
|  | The L, M, and S values for a height of 156 cm in **non-specific** LVM normative data (girls and boys in S1 Dataset): | The L, M, and S values for a height of 156 cm in **sex-specific** LVM normative data (girls in S1 Dataset): |
|  | L = - 0.2646  M = 134.3325  S = 0.1821 | L = 0.1689  M = 126.5294  S = 0.1445 |
| (2) | $z\text{-}score=\frac{\left[ \left( \frac{162.1156}{134.3325} \right)^{-0.2646}-1 \right]}{-0.2646\times0.1821}$ | $z\text{-}score=\frac{\left[ \left( \frac{162.1156}{126.5294} \right)^{0.1689}-1 \right]}{-0.1689\times0.1445}$ |
| (3) | $z\text{-}score=1.0071$ | $z\text{-}score=1.7515$ |
|  | The LVM z-score above +1.65 indicates LV hypertrophy. Application of normative data that are not sex-specific results in an underestimation of relative LVM. This causes that LV hypertrophy is not recognized. | |

**Child 2**: 16-year-old boy, 164 cm in height, with actual LVM = 179,1338g (observation number 753 in the S4 Dataset). The LVM z-score calculations are made according to equation (1), based on the respective L, M, and S values corresponding to his height, as follows:

| (1) | $z\text{-}score=\frac{\left[ \left( \frac{actual LVM}{M} \right)^{L}-1 \right]}{L\times S}$ | |
| --- | --- | --- |
|  | The L, M, and S values for a height of 156 cm in **non-specific** LVM normative data (girls and boys in S1 Dataset): | The L, M, and S values for a height of 156 cm in **sex-specific** LVM normative data (girls in S1 Dataset): |
|  | L = - 0.2802  M = 129.1153  S = 0.1807 | L = - 0.1714  M = 136.1734  S = 0.1893 |
| (4) | $z\text{-}score=\frac{\left[ \left( \frac{179.1338}{128.1153} \right)^{-0.2802}-1 \right]}{-0,2802\times0.1807}$ | $z\text{-}score=\frac{\left[ \left( \frac{179.1338}{136.1734} \right)^{-0.1714}-1 \right]}{-0.1714\times0.1893}$ |
| (5) | $z\text{-}score=1.7314$ | $z\text{-}score=1.4150$ |
|  | The LVM z-score above +1.65 indicates LV hypertrophy. Application of normative data that are not sex-specific results in an overestimation of relative LVM. This causes a false diagnosis of left ventricular hypertrophy. | |

1. Computation of LVM z-score for an individual child, from normative data, produced based on the LVM-for-height ratio adjusted with specific allometric exponents, expressed as a mean and standard deviation.

**Child 1**: 15-year-old girl, 166 cm (1.66 m) in height, with actual LVM = 162.1156 g (observation number 282 in the S4 Dataset). The LVM z-score calculations are made according to equation (6), based on the respective allometric exponents, mean LVM indexes, and standard deviation values, as follows:

| (6) | $z\text{-}score=\frac{actual LVM index-mean LVM index (normative data)}{standard deviation (normative data)}$  where, the actual LVM index stands for a ratio of the actual LVM to respective body size variable raised to a power equal to the specific allometric exponent. | |
| --- | --- | --- |
|  | **Non-specific LVM normative data;**  Table 3 in the manuscript: | **Sex-specific LVM normative data;**  Table 3 in the manuscript: |
|  | Allometric exponent = 2.6217  Mean LVM index = 37.1058  Standard deviation = 7.0667 | Allometric exponent = 2.4340  Mean LVM index = 37.1008  Standard deviation = 5.5716 |
| (7) | $z\text{-}score=\frac{\left( \frac{162.1156}{{1.66}^{2.6217}} \right)-\left( 37.1058 \right)}{7.0667}$ | $z\text{-}score=\frac{\left( \frac{162.1156}{{1.66}^{2.4340}} \right)-\left( 37.1008 \right)}{5.5716}$ |
| (8) | $z\text{-}score=0.8243$ | $z\text{-}score=1.8153$ |
|  | The LVM z-score above +1.65 indicates LV hypertrophy. Application of normative data that are not sex-specific results in an underestimation of relative LVM. This causes that LV hypertrophy is not recognized. | |

**Child 2**: 16-year-old boy, 164 cm (1.64 m) in height, with actual LVM = 179,1338g (observation number 753 in the S4 Dataset). The LVM z-score calculations are made according to equation (6), based on the respective allometric exponents, mean LVM indexes, and standard deviation values, as follows:

| (6) | $z\text{-}score=\frac{actual LVM index-mean LVM index (normative data)}{standard deviation (normative data)}$  where, the actual LVM index stands for a ratio of the actual LVM to respective body size variable raised to a power equal to the specific allometric exponent. | |
| --- | --- | --- |
|  | **Non-specific LVM normative data**   - Table 3 in the manuscript: | **Sex-specific LVM normative data;**   - Table 3 in the manuscript: |
|  | Allometric exponent = 2.6217  Mean LVM index = 37.1058  Standard deviation = 7.0667 | Allometric exponent = 2.5766  Mean LVM index = 39.7813  Standard deviation = 7.6853 |
| (9) | $z\text{-}score=\frac{\left( \frac{179.1338}{{1.64}^{2.6217}} \right)-\left( 37.1058 \right)}{7.0667}$ | $z\text{-}score=\frac{\left( \frac{179.1338}{{1.64}^{2.5766}} \right)-\left( 39.7813 \right)}{7.6853}$ |
| (10) | $z\text{-}score=1.6788$ | $z\text{-}score=1.3393$ |
|  | The LVM z-score above +1.65 indicates LV hypertrophy. Application of normative data that are not sex-specific results in an overestimation of relative LVM. This causes a false diagnosis of left ventricular hypertrophy. | |

For the computation of LVM z-score for an individual child, from the normative data produced based upon the other LVM-for-body size ratios presented in this study (Table 3 in the manuscript), the same equation (6) and procedure as above is used.
