# Supplementary material for: Left ventricular mass normalization in child and adolescent athletes must account for sex differences
Source: PLoS One. 2020 Jul 27;15(7):e0236632. doi: 10.1371/journal.pone.0236632 (PMC7384656; doi:10.1371/journal.pone.0236632)
Supplement: Attachment — Submitted filename: article revisio.docx [file pone.0236632.s011.docx]

I had the opportunity to review the study entitled “Left ventricular mass normalization in child and adolescent athletes must account for sex differences” by Hubert Krysztofiak and colleagues. In this study they analyzed 331 girls and 490 boys between 5 and 19 years old to assess the effect of sex on Left Ventricular Mass (LVM), based on echocardiography. Authors developed seven sets of the LVM normative data using different methodologies to test concordance between sex-specific and non-specific normative data, and they found that data should be developed separately for girls and boys practicing sports to avoid underdiagnosing left ventricular hypertrophy in girls and to avoid overdiagnosis in boys. The manuscript is well written and is presented in an intelligible standard English. Despite the difficulty to understand how the normative data sets have been created, the reader can understand it more easily, thanks to the explanations given step by step. Although the question of whether LVM is sex- specific or not is still open, results from this manuscript are in according to other manuscripts that have reached the same conclusion. I believe that despite the difficulty in understanding the normative data set, the methodology used to test the hypothesis has been adequate to achieve the results.

However, I would like two topics to be developed a little more extensively.

First, in the discussion section, what does the measurement of LVM provide on the determination of the thickness of the interventricular septum and posterior wall in the determination of left ventricular hypertrophy?

Second on page 21, line 21, I would like the clinical application to be exemplified in some way. For example, the determination of Z score from an individual athlete using the normative data of the supplementary material.
