# Supplementary material for: Left ventricular mass normalization in child and adolescent athletes must account for sex differences
Source: PLoS One. 2020 Jul 27;15(7):e0236632. doi: 10.1371/journal.pone.0236632 (PMC7384656; doi:10.1371/journal.pone.0236632)
Supplement: Attachment — Submitted filename: Response to Reviewers.docx [file pone.0236632.s012.docx]

March 31st, 2020

Elena Cavarretta, MD, PhD.

Academic Editor

PLOS ONE

Dear Professor Cavarretta,

According to your decision, after taking into account the concerns and criticisms addressed by the reviewers, we are submitting a revised version of the manuscript entitled " Left ventricular mass normalization in child and adolescent athletes must account for sex differences " (PONE-D-20-05310). Considering the reviewers' suggestions, we have introduced a few changes to the manuscript.

Below are detailed, point-by-point answers (written in blue) to the comments and queries from the reviewers (red). Quotes from the manuscript are italicized.

On behalf of all co-authors, sincerely yours,

Hubert Krysztofiak, MD, PhD

orcid.org/0000-0003-4567-1433

Mossakowski Medical Research Centre

Polish Academy of Sciences

5 Pawinskiego Str., 02-106 Warsaw, Poland

hkrysztofiak@imdik.pan.pl

Response to reviewers

**Reviewer #1:** I had the opportunity to review the study entitled “Left ventricular mass normalization in child and adolescent athletes must account for sex differences” by Hubert Krysztofiak and colleagues. In this study they analyzed 331 girls and 490 boys between 5 and 19 years old to assess the effect of sex on Left Ventricular Mass (LVM), based on echocardiography. Authors developed seven sets of the LVM normative data using different methodologies to test concordance between sex-specific and non-specific normative data, and they found that data should be developed separately for girls and boys practicing sports to avoid underdiagnosing left ventricular hypertrophy in girls and to avoid overdiagnosis in boys. The manuscript is well written and is presented in an intelligible standard English. Despite the difficulty to understand how the normative data sets have been created, the reader can understand it more easily, thanks to the explanations given step by step. Although the question of whether LVM is sex- specific or not is still open, results from this manuscript are in according to other manuscripts that have reached the same conclusion. I believe that despite the difficulty in understanding the normative data set, the methodology used to test the hypothesis has been adequate to achieve the results.

However, I would like two topics to be developed a little more extensively.

Q1: First, in the discussion section, what does the measurement of LVM provide on the determination of the thickness of the interventricular septum and posterior wall in the determination of left ventricular hypertrophy?

Thank you very much for the comment.

Calculated left ventricular mass is a derivative of the septal and posterior wall thickness as well as LV internal diameter. Although the use of LV wall thickness measurements to identify and monitor LV hypertrophy may seem easier in everyday clinical practice, it should be noted that the sensitivity, specificity, and prognostic accuracy of LV mass in detecting LV hypertrophy are higher than when measuring only LV wall thickness. Please, see Barbieri A, et al. Left ventricular hypertrophy reclassification and death: application of the Recommendation of the American Society of Echocardiography/European Association of Echocardiography.

We have briefly addressed this issue at the end of the Discussion section.

Q2: Second on page 21, line 21, I would like the clinical application to be exemplified in some way. For example, the determination of Z score from an individual athlete using the normative data of the supplementary material.

We have added examples of LVM z-scores calculations to the supporting information. In the supplementary file (S1 Text), the LVM z-scores calculations are shown. They picture the overestimation of relative LVM in boys and underestimation in girls when the LVM normative data that are not sex-specific are used.

The relevant information was included in the Discussion.

**Reviewer #2:** The article by Krysztofiak H. aims to assess whether the left ventricular mass (LVM) normative echocardiographic data should be developed separately for girls and boys practicing sports. By using a linear regression model, the authors demonstrated that the sex affects the relationship between LVM and body size in a large cohort of Caucasian pediatric subjects aged 5-19 years old, suggesting the claim for sex-specific LVM normative data.

In addition to the limitations already mentioned in the article, there are some concerns that, in my opinion, should be addressed by the authors in order to improve the clinical relevance of the paper:

Q3: How long did it take to enroll the subjects?

It was a retrospective study based on echocardiographic data collected between 2013 and 2018. We have added this information to the sub-section "The study participants".

Q4: The type of sport might differentially affect LV remodeling. Do you have any data on the discipline practiced by the enrolled population? Were all endurance sports?

Yes, we have information about the sport practiced by our youth athletes. Starting with the most popular, they had practiced: soccer, swimming, basketball, handball, fencing, rowing, tennis, dancing, distance running, speed skating, cycling, sailing, and martial sports like karate, taekwondo, judo, and wrestling.

As you can see, we studied a group of young athletes practicing various sports. However, since these were child and adolescent athletes, predominantly amateur, the training was focused mainly on general physical development, building the aerobic capacity, and motor skills.

We have added this information to the sub-section "The study participants".

Q5: The authors should also report the intensity (low power or high power) and duration (hours per week) of sport activity and whether the subjects were professional or amateur-level athletes.

The children and adolescents whose echocardiographic data were used in this study were athletes practicing amateur-level sport, mainly. Their training was focused primarily on general physical performance, building the aerobic capacity, and motor skills.

At the medical evaluation, we had been registering the weekly volume of training estimated as the product of the average number of training sessions per week and the average duration of a single session. We have added the information about training volume to Table 1, which presents the characteristics of the study participants.

It seems important to note that the main aim of our study was not a presentation of LVM normative data for a specific group of children and adolescents. This is a continuation of our previous works on the improvement of the methodology of LVM scaling, and these normative data, which were constructed in the present study, were used to evaluate the concordance between sex-specific and non-specific LVM normative data developed according to different methods.

Q6: Can you exclude different levels of training in the two groups?

A good question. We cannot exclude that in our study group, the level of training is different in girls and boys. Even if the volume of training would be similar, it is almost for sure that the absolute exercise intensities differ. Perhaps in the youngest, the differences are nonsignificant, but with age, the gap is growing. A comparison of world records shows it clearly. There are differences in exercise capacity between boys and girls, which cannot be explained by differences in body size. Of course, the training volume and intensity have to be adapted to the athlete exercise capacity, and in general, the absolute training workloads in girls are lower. However, in the context of the question posed in this study, these physiological differences further support the conclusions of the study that normative data for left ventricular mass should be developed separately for girls and boys.

We have addressed this issue in the sub-section "The study limitations."

Q7: Did you exclude young athletes with abnormalities on ECG suggesting cardiomyopathies but minor/ambiguous abnormalities on TTE?

Yes, certainly. Our study group consisted of healthy children and adolescents undergoing regular medical monitoring as part of the periodic preparticipation physical evaluation (PPE) at the National Center for Sports Medicine. In our country, periodic PPE is mandatory for all athletes. For children and adolescents, it is delivered as a part of the public health service. When during PPE the examining physician noticed innocent heart murmurs or suspicious electrocardiographic findings, he or she ordered echocardiography. However, as we stated in "The study participants" sub-section, "*the athletes in whom echocardiography revealed significant acquired or congenital heart diseases, affecting heart size and hemodynamics, were not included in the study*."

Q8: Do you have any electrocardiographic data about the population investigated? It would be interesting to know how many athletes had positive criteria for LVH at ECG among the two groups in contrast.

We agree it would be interesting. The electrocardiogram is an obligatory part of the medical screening of athletes, and we are collecting ECG data. However, since the aim of the present study was to assess whether the LVM normative data should be developed separately for girls and boys practicing sports, a distinct investigation is needed for such analysis.

Q9: Would it be methodologically finer to gather patients in specific age-groups?

Considering the methodology of normalization of cardiac dimensions and LVM for body size in children and adolescents, we are sure that it would not improve the quality of the study. In our study, we applied methodologies introduced by others (see, for example, Foster et al. 2008, 2013, 2016; Pettersen et al. 2008, Lopez et al. 2017) and by our team in previous works. One needs to realize that we (like many others) normalize LVM for body size. Since in healthy children, body size changes with age in a predictable way, the body size parameters, and in our opinion, especially height, can be treated as a surrogate of age. When proper normalization for body size is made in children and adolescents, there is no need for additional division on age groups. Our previous works' primary aim, as well as the present study, is to improve the methodology of cardiac size scaling for body size.

Q10: Can you provide age and sex-related LVM reference values?

Regarding this and previous question, we would like to emphasize that the main aim of our study was not a presentation of LVM normative data for the group of child and adolescent athletes. The study aimed to assess whether, for reliable evaluation of LVM in children and adolescents, left ventricular mass normative data should be developed separately for girls and boys. These normative data, which were constructed in the present study, were used to evaluate the concordance between sex-specific and non-specific LVM normative data developed according to different methods.

In our previous studies, we presented normative data for child and adolescent athletes and compared them to that shown by others:

Krysztofiak H, Małek ŁA, Młyńczak M, Folga A, Braksator W (2018) Comparison of echocardiographic linear dimensions for male and female child and adolescent athletes with published pediatric normative data. PLoS ONE 13(10): e0205459. https://doi.org/10.1371/journal.pone.0205459

Krysztofiak H, Młyńczak M, Folga A, Braksator W, Małek ŁA. Normal Values for Left Ventricular Mass in Relation to Lean Body Mass in Child and Adolescent Athletes. Pediatr Cardiol (2019) 40: 204. https://doi.org/10.1007/s00246-018-1982-9

Yet the present normative data were made with care and using a reliable methodology. The LVM normative data developed according to the LMS method are usually presented as centile curves of LVM against explanatory body size variable, similar to the so popular growth curves. It is not practiced to present the curves for a narrow age range if they were constructed for a wide age range (see Foster et al. 2013 and 2016).

For the LVM normative data developed based on the ratio of LVM to the height adjusted with (raised to the power of) specific allometric exponents, these specific exponents were determined for full age range groups (5-19); "specific" means that it is distinctive for a particular group. Thus, it would not be reasonable to present normative data for narrower age sub-groups that were selected from the main group; if so, then these sub-groups would require their own specific allometric exponents. The same for the LVM normative data developed based on the ratio of LVM to the allometrically adjusted BSA.

Eventually, only for the LVM normative data constructed based on the ratios of LVM to BSA and LVM to height raised to the power of 2.7, the age ranges normative data would be justified. However, in our previous study, we proved that BSA is not a proper explanatory variable for cardiac size scaling (Krysztofiak et al. 2019-1). In another previous study, we showed that group-specific allometric exponent should be used instead of the universal exponent of 2.7, to avoid constraints related to incomplete elimination of body size information from the normalized LVM (Krysztofiak et at. 2019-2).

Q11: Can you provide intra-observer and inter-observer analyses to guarantee for echocardiographic measurement reproducibility?

Since the study was retrospective, based on historical medical records that had been collected since 2013, the intraobserver and interobserver variability for echocardiographic measurements were not analyzed. However, all these echocardiographic measurements were performed by two experienced cardiologists in one medical center.

We have added this statement to "The study limitations" sub-section.

Q12: Mean blood pressure and heart rate values should be reported.

Thank you for that recommendation, we have added the data to Table 1, which shows the characteristics of the study participants.
