# Supplementary material for: Left ventricular mass normalization in child and adolescent athletes must account for sex differences
Source: PLoS One. 2020 Jul 27;15(7):e0236632. doi: 10.1371/journal.pone.0236632 (PMC7384656; doi:10.1371/journal.pone.0236632)
Supplement: Attachment — Submitted filename: Response to Reviewers.docx [file pone.0236632.s013.docx]

June 7th, 2020

Daniel M. Johnson, PhD.

Academic Editor

PLOS ONE

Dear Doctor Johnson,

According to your decision, after taking into account the comments addressed by the reviewers, we are submitting a revised version of the manuscript entitled "Left ventricular mass normalization in child and adolescent athletes must account for sex differences" (PONE-D-20-05310). Considering the reviewers' suggestions, we have introduced a few changes to the manuscript.

Below are detailed, point-by-point answers (written in blue) to the comments and queries from the reviewers (red). Quotes from the manuscript are italicized.

On behalf of all co-authors, sincerely yours,

Hubert Krysztofiak, MD, PhD

orcid.org/0000-0003-4567-1433

Mossakowski Medical Research Centre

Polish Academy of Sciences

5 Pawinskiego Str., 02-106 Warsaw, Poland

hkrysztofiak@imdik.pan.pl

Response to reviewers

**Reviewer #2:** The authors answered to all the raised questions and the impression is that the quality of the manuscript has been overall improved.

Some minor concerns and one question to the authors:

1) Discussion: page 20, lines 1-4, the sentence “However… sex-related differences in LVM” appears wordy and disconnected, please reprhase.

Thank you very much for the comment. As you suggested, we've rephrased this sentence as follow:

“*According to some researchers, there is no difference between boys and girls in the course of changes of LVM against LBM. They argue that since lean body mass (or fat-free mass, FFM) is the strongest determinant of LVM, the sex-related differences in LVM can be explained by the differences in LBM between boys and girls.*”

2) Discussion: page 20, lines 7-10. The sentence “But…. impedance analysis” also appears wordy. Please check the punctuation to make the sentence easier to read.

Thank you very much for the comment. As you suggested, we've rephrased this sentence as follow:

“*Since LBM cannot be measured directly, advanced indirect methods such as double X-ray absorptiometry, computed tomography, magnetic resonance imaging, or bioelectrical impedance analysis are required for reliable measurements*.”

3) This analysis has been performed on a sportive pediatric population, though not professional athletes have been considered. Do you think that your results should be applied to all pediatric populations regarding the participation in sport? Or do you think that the different kind and amount of sport performed by the two different sexes might have in some manner influenced the results? This question is raised while evaluating a young patient with a suspicion of cardiomyopathy, especially in they “grey” zone. Should we correct the LVM for sex even in this population? The authors should address this topic in the discussion to give further clinical impact to your work.

The children and adolescents whose echocardiographic data were used in this study were athletes practicing mostly amateur-level sport. An athletic population is considered as the special one because regular exercise contributes to increasing cardiac size, which is an adaptive response causing increased exercise capacity. As the training volume and intensity have to be adapted to the athlete exercise capacity, the absolute training workloads in girls are lower than in boys. Therefore, we cannot exclude that the different intensity of training in girls and boys additionally contributed to the fact that for a given body size LVM in boys was higher than in girls.

Thus, we agree that the specificity of the population examined in this study may have "in some manner influenced the results," but this is not a limitation; it is an advantage. The group selection allowed us to demonstrate that there are differences in cardiac size between boys and girls, which cannot be explained by differences in body size.

At the same time, we know that regardless of whether they practice sports or not, there are differences in exercise capacity between boys and girls, which cannot be explained by differences in body size. In general, the exercise capacity in boys is greater than in girls. Perhaps in the youngest, the differences are nonsignificant, but with age, the gap is growing. Athletic training might only augment the difference in exercise capacity between boys and girls within the population. The same goes for cardiac size.

Therefore, we are convinced that for all pediatric populations, one should use LVM normative data that were developed separately for girls and boys.

We have introduced relevant information to the discussion and the study limitations sections.
