# Supplementary material for: Left ventricular mass normalization in child and adolescent athletes must account for sex differences
Source: PLoS One. 2020 Jul 27;15(7):e0236632. doi: 10.1371/journal.pone.0236632 (PMC7384656; doi:10.1371/journal.pone.0236632)
Supplement: Attachment — Submitted filename: Response to Reviewers.docx [file pone.0236632.s014.docx]

July 5th, 2020

Daniel M. Johnson, PhD.

Academic Editor

PLOS ONE

Dear Doctor Johnson,

According to your decision, after taking into account the comments addressed by the reviewer, we are submitting a revised version of the manuscript entitled "Left ventricular mass normalization in child and adolescent athletes must account for sex differences" (PONE-D-20-05310).

Considering the reviewer's suggestions, we have introduced a few changes to the manuscript. Below is the answer (written in blue) to the comments from the reviewer (red).

On behalf of all co-authors, sincerely yours,

Hubert Krysztofiak, MD, PhD

orcid.org/0000-0003-4567-1433

Mossakowski Medical Research Centre

Polish Academy of Sciences

5 Pawinskiego Str., 02-106 Warsaw, Poland

hkrysztofiak@imdik.pan.pl

Response to reviewer

**Reviewer #2:**

The authors have adequately rephrased the sentences as suggested (point 1 and 2).

However, the new section added to the discussion (point 3 – "Since the study has been performed … for cardiac size.") needs rephrasing and implementation; specifically, a) informal registry ("..we are convinced that yes, … the same goes for …"), b) incorrect tenses or grammar errors ("..as the special one because regular exercise contribute to …. the gap is growing."), c) be more precise when stating that in the youngest differences in exercise capacity are non-significant, from which age should we consider that gap significant? Please, clarify the relationship between exercise capacity and LVM in non-athletes and how this information impacts on your issue.

Thank you, we appreciate your comments. The errors you pointed out should not happen. We have rewritten this section and introduced additional information addressing substantive issues you raised.

This section now reads as follows:

"The study participants were child and adolescent athletes. The athletic population may be considered special because regular exercise contributes to an increase in cardiac size [15], and specific LVM normative data are recommended for child and adolescent athletes [20]. A question may thus arise as to whether the results of the study should be applied to all children and adolescents.

The increase in cardiac size in response to exercise is an adaptive phenomenon linked to the improvement of exercise capacity. Not only athletes but generally all healthy children, both boys, and girls, have higher LVM when their exercise capacity is higher [50]. However, boys have higher exercise capacity than girls of the same age, and this is true for both athletic and non-athletic populations [51]. Perhaps in the youngest, the difference is not seen, but after the age of about twelve, it becomes significant [52]. The same pattern is observed in the case of LVM. Before puberty, LVM in healthy boys and girls is similar, and at puberty, the difference between boys and girls becomes evident, with higher LVM in boys [53].

Thus, higher exercise capacity is associated with higher LVM, and within comparable groups of healthy children, especially adolescents, boys have higher exercise capacity and LVM than girls. That is not solely specific to athletes. Normative data for LVM should be developed separately for boys and girls for all children and adolescents, regardless of whether they practice sports or not."
